# Biofabrication of an *in-vitro* bone model for Gaucher disease

**DOI:** 10.1088/1758-5090/acf95a

**Published:** 2023-09-22

**Authors:** Dishary Banerjee, Margarita M Ivanova, Nazmiye Celik, Myoung Hwan Kim, Irem Deniz Derman, Renuka Pudi Limgala, Ibrahim T Ozbolat, Ozlem Goker-Alpan

**Affiliations:** 1 Engineering Science and Mechanics Department, Penn State University, University Park, PA, United States of America; 2 Department of Medicine, Division of Cardiology, University of California, San Diego, La Jolla, CA, United States of America; 3 Lysosomal & Rare Disorders Research & Treatment Center—LDRTC, Fairfax, VA, United States of America; 4 Department of Biomedical Engineering, Pennsylvania State University, University Park, PA, United States of America; 5 The Huck Institutes of the Life Sciences, Pennsylvania State University, University Park, PA, United States of America; 6 Materials Research Institute, Pennsylvania State University, University Park, PA, United States of America; 7 Department of Neurosurgery, Pennsylvania State College of Medicine, Hershey, PA, United States of America; 8 Medical Oncology, Cukurova University, Adana, Turkey; 9 Biotechnology Research and Application Center, Cukurova University, Adana, Turkey

**Keywords:** Gaucher disease, 3D bioprinting, bone model, osteoblastic–osteoclastic co-differentiation

## Abstract

Gaucher disease (GD), the most prevalent lysosomal disorder, is caused by *GBA1* gene mutations, leading to deficiency of glucocerebrosidase, and accumulation of glycosphingolipids in cells of the mononuclear phagocyte system. While skeletal diseases are the leading cause of morbidity and reduced quality of life in GD, the pathophysiology of bone involvement is not yet fully understood, partly due to lack of relevant human model systems. In this work, we present the first 3D human model of GD using aspiration-assisted freeform bioprinting, which enables a platform tool with a potential for decoding the cellular basis of the developmental bone abnormalities in GD. In this regard, human bone marrow-derived mesenchymal stem cells (obtained commercially) and peripheral blood mononuclear cells derived from a cohort of GD patients, at different severities, were co-cultured to form spheroids and differentiated into osteoblast and osteoclast lineages, respectively. Co-differentiated spheroids were then 3D bioprinted into rectangular tissue patches as a bone tissue model for GD. The results revealed positive alkaline phosphatase (ALP) and tartrate-resistant ALP activities, with multi-nucleated cells demonstrating the efficacy of the model, corroborating with gene expression studies. There were no significant changes in differentiation to osteogenic cells but pronounced morphological deformities in spheroid formation, more evident in the ‘severe’ cohort, were observed. Overall, the presented GD model has the potential to be adapted to personalized medicine not only for understanding the GD pathophysiology but also for personalized drug screening and development.

## 1. Introduction

Gaucher disease (GD), the most common lysosomal storage disorder, is caused by a deficiency of the enzyme glucocerebrosidase (GCase). Accumulation of the substrate, glucosylceramide (GC), most notably in macrophages, leads to diverse phenotypic effects including hepatosplenomegaly, skeletal and nervous system involvement, and immune dysregulation with abnormalities in the differentiation of the mononuclear phagocyte lineage [1]. The three GD clinical types are distinguished based on the involvement of the central nervous system (CNS) and the rapidity and extent of the progression of the neurological disease. GD type 1 disease (GD1), also called non-neuronopathic GD, lacks the primary CNS disease. Neuronopathic forms (GDn) are characterized by onset from infancy to early childhood, either with rapid progression of neurological symptoms (type 2), or with severe systemic manifestations and chronic neurologic involvement (type 3) [2].

Bone-related problems represent the principal unmet medical need in GD as all patients with GD are at risk of complications due to bone disease. 75% of GD patients develop skeletal complications, including bone remodeling defects, osteopenia, osteoporosis, abnormal vertebral remodeling, Erlenmeyer flask deformity, marrow infiltration, avascular necrosis, and osteolysis [3, 4]. Untreated pediatric patients in the International Collaborative Gaucher Group Gaucher Registry have exhibited skeletal manifestations similar to that of adults, Erlenmeyer flask deformity (81%), pain (27%), and bone crises (9%) [5]. In children, one of the GD presentations is growth retardation [2]. While in the neuropathic form of GD (type 3), bone pain and avascular necrosis are rare, progressive kyphoscoliosis is associated with severe systemic involvement [6]. However, the underlying cellular/molecular basis of bone development and related complications in GD has yet to be explained and there are no known specific biomarkers associated with a given bone pathology, that could assist therapeutic planning and clinical management in different GD clinical types and age groups.

Cells of monocyte lineage that play essential roles in bone metabolism are primarily affected in GD. GD bone pathology ranges from osteonecrosis to bone density and bone growth abnormalities. Reduced bone density and replacement of normal fatty marrow suggest that the downstream pathways associated with glycosphingolipid catabolism may affect both hematopoiesis and the balance of osteoblast and osteoclast numbers and activity. Chronic immune stimulation promotes an imbalance between bone formation and breakdown resulting in disordered trabecular and cortical bone modeling, cortical bone thinning, and fractures. Monocytes/macrophages play important roles in bone morphogenesis and remodeling. Monocytes have the ability to differentiate into osteoclasts under suitable microenvironments and produce several osteogenic factors, which may influence the differentiation of osteoblasts [7].

Chronic immune stimulation of GD macrophages can damage bone tissue through the secretion of cytokines (monocyte chemo-attractant proteins (MCP-1), tumor necrosis factor (TNF)-*α*, interleukin (IL-6, IL10, and IL4) [1, 4]. Moreover, locally increased cytokines in bone stimulate the production of osteoclast precursors, resulting in imbalances in bone remodeling to favor resorption over formation, leading to osteopenia or osteoporosis [8, 9]. Important components that regulate bone remodeling are receptor activator of NF-kB ligand (RANKL), osteoprotegerin, dickkopf-1 (DDK-1) and sclerostin [10, 11]. The RANKL/RANK signaling pathway is essential for regulating bone development and bone resorption [12, 13]. Expressed by osteoblasts, RANKL activates osteoclast differentiation and maturation, favoring bone resorption [14]. In GD, RANKL is elevated and correlates with osteopenia. Moreover, the anti-RANKL antibody, Denosumab, has been used for GD patients with osteoporosis to prevent osteoclast development through RANKL inhibition.

Osteoclast biomarker, tartrate-resistant acid phosphatase (TRAP) is an enzyme coded by acid phosphatase 5 (ACP5). Two isoforms of TRAP circulate in the blood: TRAP5a secreted from macrophages and dendritic cells, and TRAP5b secreted from osteoclasts. TRAP5b is a marker of osteoclast activity and an indicator of bone resorption [15]. TRAP5b, highly expressed in osteoclasts and is shown to be responsible for bone resorption in GD [10] and correlated with osteopenia and osteoporosis in GD patients [14]. Moreover, TRAP5b positively correlates with clinical biomarkers of GD pathology: CCL18, glucosylsphingosine (lyso-Gb1), and chitotriosidase [14, 16].

Activators of bone resorption, DKK-1 and sclerostin, are elevated in GD, and sclerostin is associated with reduced bone mineral density, bone pain, bone marrow infiltration, and EM flask deformity [17]. Pharmacological inhibition of sclerostin and DKK-1 by monoclonal antibodies is one of the novel therapy for osteoporosis that is capable to promote new bone tissue growth [18–20]. Overall, while monoclonal antibodies to RANKL, Sclerostin, and DKK-1 for the treatment of osteoporosis are approved or in clinical trials, there is still a lack of knowledge regarding how to use these drugs effectively and which biomarkers must influence therapy selection. Development of new 3D models, which represent osteoblast and osteoclast differentiation, and mimics bone development and bone microenvironment could help assess the efficiency of monoclonal antibody therapies to treat bone manifestation efficiency of monoclonal antibody therapies to treat bone manifestation in patients with GD. Previous *in vitro* models for mimicking GD only used macrophages [21] or osteoclasts [22] or osteoblasts [23] individually which are far from the recapitulation of the D pathology.

In this study, we aim to bridge the gap by proposing an *in-vitro* bone model for studying GD using peripheral blood-derived monocytes (PBMCs) derived from patients with GD and cocultured with human mesenchymal stem cells (hMSCs) as precursors of osteoclasts and osteoblasts, respectively, and their bioprinting to develop a physiologically-relevant system. Using aspiration-assisted freeform bioprinting, we henceforth demonstrated the successful assembly of hMSC and cocultured (with monocytes-derived osteoclasts) spheroids and their respective osteogenic and osteoclastogenic differentiation for bone tissue formation. Since osteoclasts also play a significant role in bone remodeling [24–26], to the best of our knowledge, we also for the very first time demonstrated the incorporation of GD patient-derived PBMC-derived osteoclasts into hMSCs to form heterocellular spheroids and investigate their role in self-assembly and consequently in formation of bone tissue. Our results presented relevant phenotypic and genotypic skeletal deformities in GD patients compared to the healthy ones arising majorly from abnormal osteoclast-related gene expression, showing the potential of using this heterotypic bioprinted model for investigating patho-physiology in GD patients.

## 2. Materials and methods

### 2.1. Subjects: clinical and bone pathology data

Six GD patients in the age group 29–66 years and three healthy controls participated in the study. Participants (*n* = 3) were further categorized into the following three groups, including GD-severe and GD-mild cohorts, and the non-GD control (the normal healthy cohort with no bone complications). A written informed consent form to collect and analyze their data was obtained from all patients. Ethics committees and data protection agencies approved the clinical protocol at all participating sites (Western Institutional Review Board, WIRB # 20131424). Detailed medical history and bone pathology characteristics were presented in table 1.

**Table 1.** Individual medical history with bone pathology characteristics (HSM: hepatosplenomegaly, EM: Erlenmeyer flask deformity, ERT: enzyme replacement therapy, SRT: substrate reduction with therapy with Eliglustat tartrate).

no	GBA genotype	Age	Sex	Clinical presentation	Bone disease	Treatment status/years	Chito mol hr^−1^ ml^−1^	Lyso Gb1 ng ml^−1^	CCL18 ng ml^−1^
Non-GD control									
1	WT	47	F	NA	None	None	NA	N/D	NA
2	WT	44	M	NA	None	None	NA	N/D	NA
3	WT	37	F	NA	None	None	NA	N/D	NA
GD mild									
4	L444P/L444P	29	F	No GD family history	None	ERT/27	34	7.0	105.0
5	N370S/N370S	51	F	HSM Borderline platelets	Mild marrow replacement EM deformity	ERT/12	52	2.7	60.9
6	N370S/ N370S	30	F	HSM Low platelets	Osteopenia Mild marrow replacement EM deformity	ERT/5	215	7.9	54.5
GD severe									
7	L444P/R493C	45	F	Severe HSM, splenectomy, multiple AVN	Osteoporosis Bilateral hip replacement, extensive cystic/lytic changes Extensive marrow involvement with chronic scarring and marrow infarcts	ERT/30 ERT + SRT/1	530	82	178.5
8	R48Q/L444P	34	M	Severe HSM Very low platelets	Osteopenia Chronic marrow infarcts Extensive marrow scarring EM deformity	ERT/7 SRT/1	1022	11	1087.9
9	N370/N370	66	M	Severe HSM Low platelets	Osteoporosis Chronic marrow infarcts and scarring EM deformity	ERT/11 SRT/11	44	1.5	56.1

### 2.2. Isolation, purification, and culture of PBMCs

PBMCs were purified from blood samples obtained from patients with GD using Lymphoprep™ reagent and SepMate™ tubes (Stemcell Technologies, Vancouver, Canada). Lymphoprep™ was added to the lower compartment of the SepMate tube. Phosphate buffered saline (PBS) containing 2% fetal bovine serum (FBS) was mixed with blood in a 1:1 ratio and then layered on top of Lymphoprep™ following the manufacturer’s protocol. Samples were centrifuged for 20 min at 800 × g at 18 °C with the brake off. The PBMC layer was removed carefully after discarding the upper plasma layer, washed three times with PBS, and centrifuged at 300 × g for 8 min at room temperature between each wash. Isolated PBMCs were incubated in 5% CO_2_ in phenol red-free Roswell Park Memorial Institute (RPMI) media with 10% FBS.

### 2.3. Culture of commercial THP-1

THP-1 cells (TIB-202; American Type Culture Collection (ATCC), Manassas, VA) were cultured in a suspension culture in pre-warmed ATCC-formulated RPMI-1640 medium, supplemented with 10% FBS and 0.05 mM 2-mercaptoethanol. When the cells reached a density of 1 million cells per ml, they were split in a 1:6 subculture ratio and used from passages 3 through 7.

### 2.4. Fabrication of heterocellular spheroids

hMSCs (commercially obtained from Rooster Bio (Frederick, MD)) were cultured in hMSC growth media (R&D Systems, MN). After reaching 90% confluency, hMSCs were trypsinized and centrifuged at 300 × g for 5 min. The media was discarded, and the pellet was resuspended in fresh hMSC growth media for forming spheroids. hMSC were used from passages 3 through 5. To obtain the required density of ∼1 × 10^6^ cells per ml, PBMCs were added to a pre-warmed ATCC-formulated RPMI-1640 medium, supplemented with 10% FBS and 0.05 mM 2-mercaptoethanol. The suspension media was collected and centrifuged. An osteoclast differentiation media was made from RPMI 1640 supplemented with 40 ng ml^−1^ phorbol 12-myristate 13-acetate (PMA, Abcam, Cambridge, UK), 10 ng ml^−1^ receptor activator of nuclear factor-kappa ß ligand (RANKL, Abcam), 10% FBS, antibiotic/antimycotic, and non-essential amino acids (Gibco, Waltham, MA). A cocktail media for differentiating hMSCs and PBMCs respectively into osteoblasts and osteoclasts was prepared by mixing the osteoblast differentiation media with osteoclast differentiation media at a ratio of 2:1. hMSCs in a previously optimized ratio of 2:1 hMSC:PBMC [27] was resuspended with the PBMCs in the cocktail media and pipetted into each well of cell-repellent U-bottom 96-well plates (Greiner Bio-One, Kremsmunster, Austria) for formation of heterocellular spheroids with ∼15 000 cells per well to attain a size of 400 *µ*m. Using the cocktail media, spheroids were cultured at 37 °C under a humidified atmosphere of 5% CO_2_ upto 4 weeks The cell medium was changed every 2–3 d. We used three groups of spheroids derived from different GD patients as hMSC:PBMC (healthy control), hMSC:PBMC (GD-mild) and hMSC:PBMC (GD-severe) for understanding the effects of GD on spheroid formation and differentiation. The hMSCs-only group was used as a control.

### 2.5. Preparation of alginate (Alg) microgels as a support bath

The equipment used to prepare Alg microgels was sterilized with 70% ethanol and ultraviolet (UV) light for 30 min. Sodium alginate (#71238, Sigma Aldrich, St. Louis, MO) was dissolved in ultra-purified water at 0.5 w/v at room temperature. The homogeneously mixed Alg solution was crosslinked by adding dropwise into 4% anhydrous calcium chloride (CaCl_2_, ⩾97%, Sigma Aldrich) using a dropping funnel (1000 ml, Eisco Labs, Victor, NY), in which the flow rate of droplets was controlled by a stopcock (maximized flow rate until single droplets were maintained). 30 min after crosslinking, the crosslinked Alg beads were collected. To remove CaCl_2_ solution and uncrosslinked Alg residues, the beads were washed thrice with ultra-purified water. A single speed commercial blender (E8100, Waring Commercial, Stamford, CT) with a blending container (E8485, Eberbach Corp., Van Buren Charter Township, MI) was set at 22 000 rpm for 30 min at 4 °C in order to blend Alg beads to obtain Alg microgels. The resultant microgels was then divided into 50 ml conical tubes and centrifuged at 2000 × g for 5 min.

### 2.6. Aspiration-assisted freeform bioprinting

For aspiration-assisted freeform bioprinting, a modified in-house custom-made bioprinting system [27, 28], was utilized. The bioprinting setup had a nozzle, spheroid reservoir, and placement area. The placement area had a fixed 35 Ø Petri dish. A 3D printed pocket was added to the Petri dish to hold Alg microgels during bioprinting. The pocket chamber (15 × 15 × 2 mm^3^) was fabricated using an Ultimaker 3 (Utrecht, Netherlands). The 3D printed pocket was sterilized with 70% ethanol, followed by exposure to UV for 1 h. Loading of Alg microgels into the square pocket was performed before bioprinting. Alg microgels (with an average size of 30 *µ*m) was used to provide support for spheroids until they fused [27]. In this study, alginate microgels offered the following advantages—they were easily removable after tissue fusion, biologically inert and do not support spheroid adhesion, and biocompatible and non-toxic, and helped in oxygen, nutrient and waste exchange through the porous gel.

Three microscopic cameras were installed to visualize the bioprinting process in real-time for top, bottom, and side views. Post 24 h of cell seeding, fabricated spheroids were transferred into the reservoir. To aspirate spheroids, a straight 27G stainless-steel nozzle (Nordson, Westlake, OH) was utilized and 70 mmHg aspiration pressure was applied. hMSC and cocultured heterocellular spheroids were aspirated individually and then gently placed in desired positions in Alg microgels at a bioprinting speed of 2.5 mm s^−1^ to generate rectangular tissue constructs. For osteogenically-inducted constructs, hMSCs-only and 2:1 hMSC:PBMC (healthy cohort, GD-mild and GD-severe) groups were cultured in their respective differentiation media as mentioned earlier. The bioprinting process was indeed a time-consuming process, in which aspiration of a single spheroid and then its precise placement took approximately 30 s, so the formation of the bioprinted structures takes a couple of minutes. We are currently developing an advanced system to improve the speed and efficiency of the process to facilitate high-throughput bioprinting of spheroids. Post 5 d of incubation, Alg microgels were removed (depending on spheroid fusion) by adding 4% (w/v) sodium citrate (Sigma Aldrich) for 15 min as a lyase. Bioprinted constructs were washed three times with Dulbecco’s Phosphate Buffered Saline (DPBS, Corning, Corning, NY) and cultured with the relevant differentiation media for 23 d.

### 2.7. Alkaline phosphatase (ALP) and acid phosphatase (AP) enzyme activity

Respective differentiation of hMSCs and PBMCs into osteoblasts and osteoclasts were quantified at Days 14 and 28 using the alkaline (Abcam) and AP assay kit (BioVision, Milpitas, CA) following the protocol provided by the manufacturers. Briefly, at the specified time points, both the differentiating single spheroids and bioprinted constructs were collected and homogenized vigorously using a homogenizer. Following homogenization, we added 400 *µ*l of buffer solution and centrifuged it at 13 000 rpm for 5 min to remove non-cellular residues. We then collected 80 *µ*l of the supernatant (in triplicates) in a 96-well plate, mixed them with 5 *µ*l of 5 mM p-nitrophenyl phosphate and incubated for 1 h at room temperature. 20 *µ*l of a manufacturer-provided stop solution was used to stop the reaction, and the optical density was measured in triplicates at 405 nm using a spectrophotometer (Bio-Tek, Winooski, VT). ALP and AP enzymes provided by the manufacturers were used to calculate the respective enzyme activity from the measured optical density.

### 2.8. Gene expression using quantitative real-time polymerase chain reaction (qRT-PCR)

Evaluation of the osteoblast- and osteoclast-related gene expression profiles in four different groups (hMSC-only) and hMSC:PBMC (healthy cohort, GD-mild and GD-severe) for the single spheroids and bioprinted constructs at Days 14 and 28 was conducted using qRT-PCR. Samples were homogenized in TRIzol reagent (Life Technologies, Carlsbad, CA) on Day 28. PureLink RNA Mini Kit (ThermoFisher) was used to isolate the total RNA from samples according to the manufacturer’s protocol. A Nanodrop (Thermo Fisher Scientific) was used for measuring RNA concentration, reverse transcription was performed using AccuPower^®^ CycleScript RT PreMix (BIONEER, Daejeon, South Korea) following the manufacturer’s instructions, and gene expression was analyzed quantitatively with SYBR Green (Thermo Fisher Scientific) with the help of a QuantStudio 3 PCR system (Thermo Fisher Scientific). Osteoblast- and osteoclast-related genes were tested including bone morphogenic protein-4 (BMP-4), bone sialoprotein (BSP), Cathepsin K (CTSK), osteoclast-associated immunoglobulin-like receptor (OSCAR) and calcitonin receptor. Expression levels for each gene were normalized to glyceraldehyde 3-phosphate dehydrogenase (GAPDH). The fold-change of commercial THP-1 on Day 1 was set as 1-fold, and values in all groups were normalized with respect to that group. The reader is referred to table 2 for the gene sequences.

**Table 2.** Primers of the measured mRNA for qRT-PCR.

Gene	Forward Primers	Reverse Primers
BMP-4	TAG CAA GAG TGC CGT CAT TCC	GCG CTC AGG ATA CTC AAG ACC
BSP	AAC GAA GAA AGC GAA GCA GAA	TCT GCC TCT GTG CTG TTG GT
CTSK	CCG CAG TAA TGA CAC CCT TT	GCA CCC ACA GAG CTA AAA GC
OSCAR	GCT TCA TAC CAC CCT AAG CC	AAA GTC CAA ATC TCC AAG CG
Calcitonin receptor	TGG TGC CAA CCA CTA TCC ATG C	CAC AAG TGC CGC CAT GAC AG
GAPDH	ATG GGG AAG GTG AAG GTC G	GGG GTC ATT GAT GGC AAC AAT A

### 2.9. CCL18 activity by enzyme-linked immunosorbent assay (ELISA)

CCL18 activity was measured in 3D bioprinted constructs over the course of experiments. Venous blood samples were collected in ethylenediaminetetraacetic acid vacutainer tubes [14]. Plasma levels of CCL18 were measured using a commercially available human CCL18 ELISA Kit (Abcam). The optical density was read at 450 nm using a spectrophotometer (FilterMax 5, Molecular Devices, San Jose, CA). Sensitivity of the ELISA kit was 0.55 pg ml^−1^ and the range was determined to be 1.53–100 pg ml^−1^, as provided by the manufacturer.

### 2.10. Statistical analysis

All data were presented as mean ± standard deviation and analyzed by Minitab 17.3 (Minitab Inc., State College, PA). Analysis of multiple comparisons was performed by one-way analysis of variance (ANOVA) followed by post-hoc Tukey’s multiple-comparison test to determine the individual differences among the groups. For statistical analysis of ALP and AP activity to compare results against the hMSC-only group and commercial THP-1, two-way ANOVA was performed, followed by posthoc Tukey’s multiple-comparison test to determine the individual differences.

## 3. Results and discussion

### 3.1. Characterization of hMSC:PBMC spheroids

Bone metabolism is a dynamic process, mutually balanced by osteoblast-mediated bone formation and osteoclast-mediated bone resorption [24]. Most studies demonstrating *in-vitro* bone models concentrate on hMSCs or osteoblasts derived from hMSCs with marginal or often no consideration of the presence of osteoclasts in bone [29, 30]; however, osteoclasts are the cells which initiate bone modeling and are known to regulate osteoblast precursors to form bone [31]. In the literature [27], 2:1 combination of hMSC:THP-1 demonstrated favorable outcomes, such as expression of both osteoclast- and osteoblast-related genes. However, the use of THP-1—a spontaneously immortalized monocyte-like cell line, derived from the peripheral blood of acute monocytic leukemia (M5 subtype) creates an obvious approximation [32]. It has been now well documented that primary monocytes respond far more to lipopolysaccharides with higher expression of cluster of differentiation (CD)-14, and alteration in IL-8 expression compared to THP-1 [33]. Since GD is characterized by accumulation of GC in lysosomes and the secretion of inflammatory cytokines, it becomes more intuitive to develop an *in-vitro* bone model for GD using primary monocytes instead of this immortalized simplified model cells.

**Figure 1.**
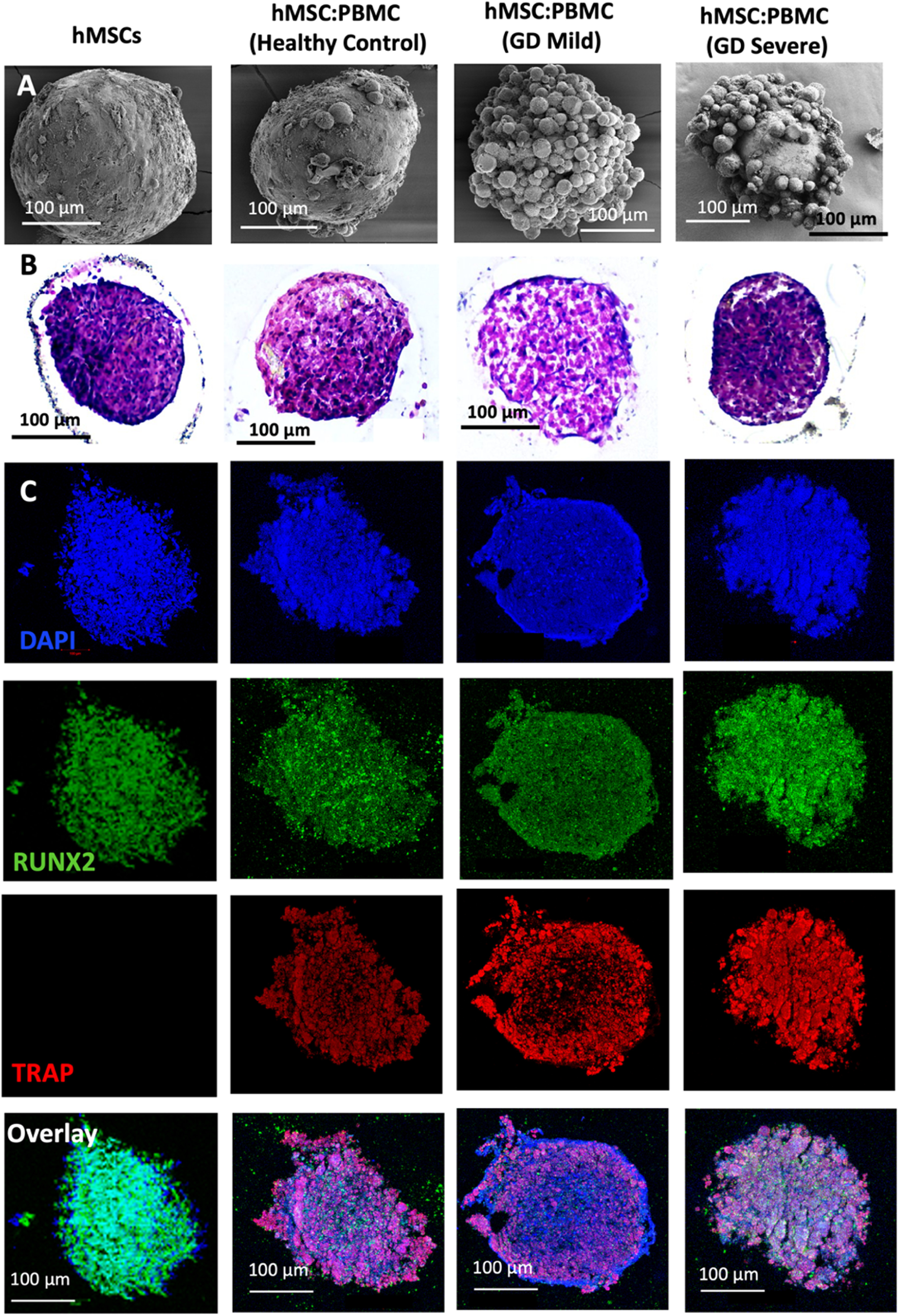
Heterocellular spheroids (2:1 hMSC:PBMC) for bone tissue fabrication as a GD model at Day 14. (A) SEM, (B) H&E, and (C) immunostaining for RUNX2 (green) for hMSCs differentiating into osteogenic cells and TRAP (red) for PBMCs differentiating into osteoclastogenic cells.

**Figure 2.**
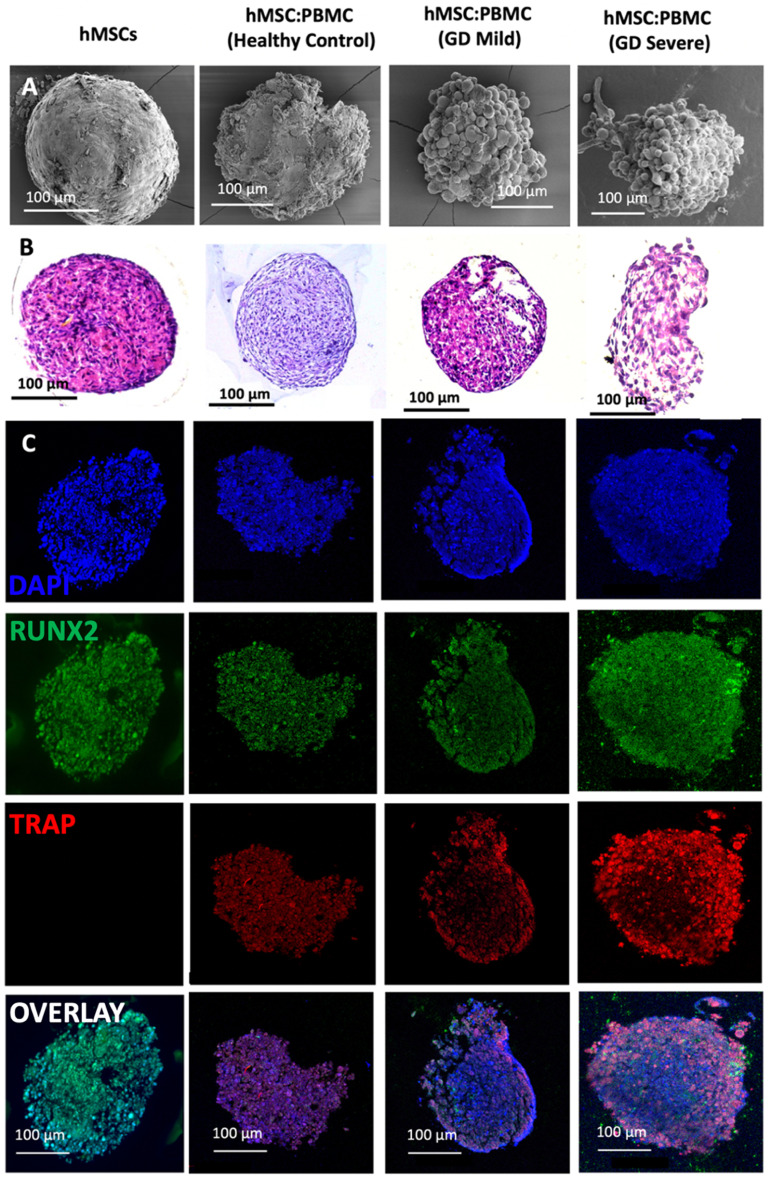
Heterocellular spheroids (2:1 hMSC:PBMC) for bone tissue fabrication as a GD model at Day 28. (A) SEM, (B) H&E and (C) immunostaining for RUNX2 (green) for hMSCs differentiating into osteogenic cells and TRAP (red) for PBMCs differentiating into osteoclastogenic cells.

In this study, we used GD patient-derived monocytes to differentiate them into osteoclasts while they were under a coculture with commercially-obtained hMSCs. A previously optimized cell ratio of 2:1 hMSC:PBMC [27] was used to fabricate the *in-vitro* bone model for GD. Cells were observed to coalesce into single spheroids and compacted over 24 h of culture. After 24 h, spheroids were cultured in differentiation media (composed of osteoblast and osteoclast differentiation media mixed in 2:1 ratio) for 28 d. The spheroids were investigated for histological characterization at Days 14 and 28. hMSC-only spheroids were used as a control. SEM micrographs (figures 1(A) and 2(A)) demonstrate a spherical morphology in all groups at Days 14 and 28. The mean diameter of hMSC-only, 2:1 hMSC:PBMC (GD healthy cohort, GD-mild and GD-severe) was determined to be 394 ± 15, 413 ± 22, 430 ± 22 and 464 ± 42 *µ*m (Day 14) and 376 ± 12, 397 ± 17, 432 ± 12 and 459 ± 30 *µ*m (Day 28), respectively (figure S1). All groups were noted to progressively undergo spheroid compaction over the course of culture. Larger circular cells were observed to accumulate at the periphery of the PBMC-involved spheroids (with more accumulation noted with increased GD severity). This may be attributed to the migration of the osteoclasts to the periphery of the spheroids because of their lower expression of E-cadherin compared to their hMSC counterparts [34]. It is also notable mentioning that the PBMC-only group was considered as a positive control group in our preliminary experiments but were observed to form only loose aggregates without any compaction or formation of single spheroids, also corroborating our hypothesis of lower E-cadherin secretion, which is inevitably important for spheroid formation [35]. H&E staining images (figures 1(B) and 2(B)) confirmed evenly distributed cells with no significant signs of necrosis in the core when comparing the number of PBMCs in the heterocellular spheroids. Significant histological difference was not observed between the hMSC and healthy cohort groups, with presence of larger multi-nucleated cells resembling osteoclasts in the healthy cohort compared to the hMSC group. hMSC and hMSC:PBMC healthy cohort spheroids exhibited denser and more uniform cytoskeletal organization as compared to GD patient-derived spheroids, which exhibited lesser cellular density and empty space at the core with the GD severe exhibiting a high nuclear content with minimal cytoplasm. Morphological deformations were noted in the heterocellular spheroids with increase in GD severity of patients especially at Day 28 (figure 2(B)) compared to Day 14, which could be related to phenotypic skeletal deformities in the GD severe patients. In the GD mild group, notably more purplish smaller abundant nuclei structures in H&E images were noted with abundant fibrillary crumbled paper-like cytoplasm, similar to a case reported in a young male [36]. In the GD severe group, whereas several larger nuclei were noted with very low amounts of cytoplasm and visible deformities in the spheroid development. Immunostaining images (figures 1(C) and 2(C)) revealed the presence of both osteoclasts (TRAP) and osteoblasts (RUNX2) in heterocellular spheroids, whereas the hMSC-only spheroids were positive to RUNX2. The more expression of TRAP towards the periphery of spheroids, specifically in GD-involved groups, supported SEM micrographs that the osteoclasts accumulate more towards the periphery of spheroids, whereas the core was primarily occupied by hMSC-derived osteoblasts. Similar morphological deformities were noted in the GD-involved groups as was observed through SEM or H&E images, especially at Day 28.

**Figure 3.**
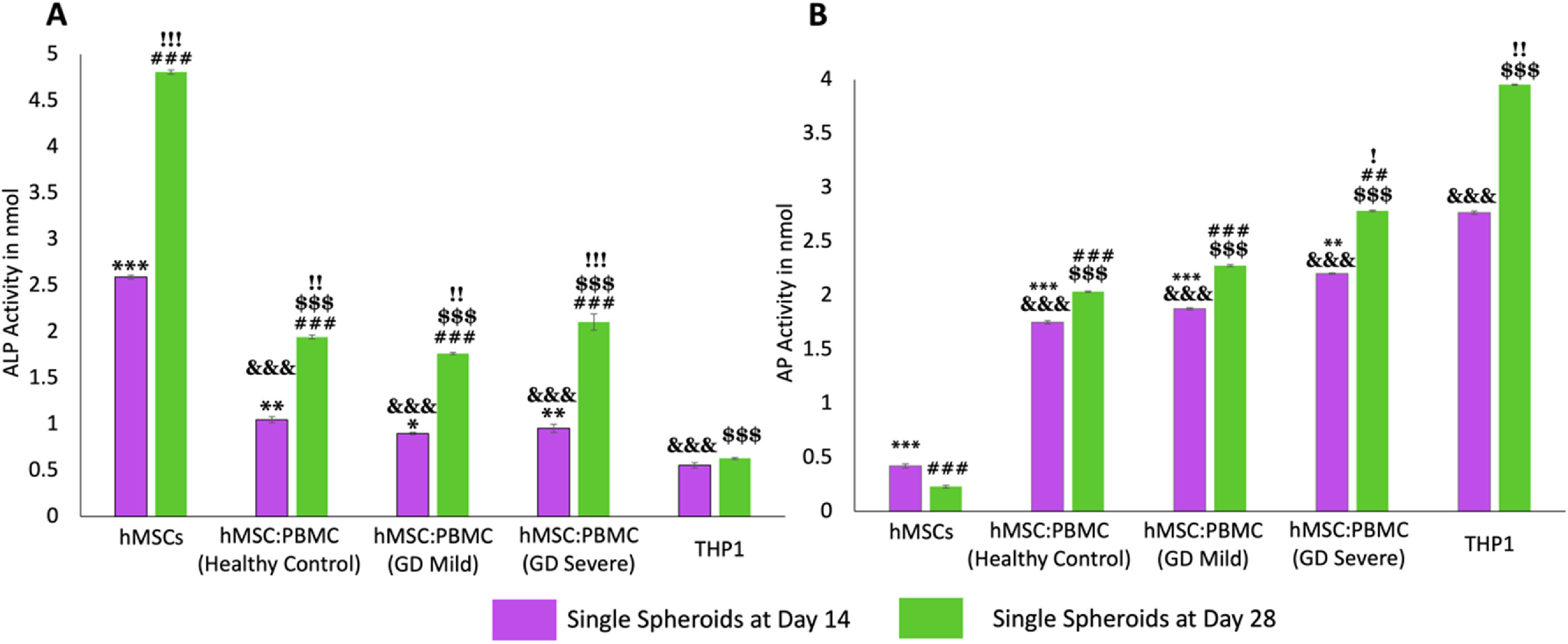
Enzymatic assays for hMSC:PBMC spheroids at Days 14 and 28. (A) ALP enzyme activity for hMSCs differentiating into osteoblasts and (B) the AP activity for PBMCs differentiating into osteoclasts. Data were presented as mean ± s.e.m (*n*= 4; *: compared to THP-1 (Day 14), p* < 0.05, p** < 0.01, p*** < 0.001; #: compared to THP-1 (Day 28), *p^#^* < 0.05, *p* ^##^< 0.01, *p* ^###^< 0.001; &: compared to hMSCs (Day 14) *p* ^&^< 0.05, *p* ^&&^< 0.01, *p* ^&&&^< 0.001; $: compared to hMSCs (Day 28), *p* ^$^< 0.05, *p* ^$$^ < 0.01, *p* ^$$$^ < 0.001; !: compared to Day 14, *p* ^!^ < 0.05, *p* ^!!^ < 0.01, *p* ^!!!^ < 0.001).

We also investigated ALP and AP enzyme activities in heterocellular spheroids to demonstrate the respective differentiation of hMSCs and PBMCs into osteoblasts and osteoclasts, respectively. Enzyme ALP is an important serum analyte, whose activity often corresponds to active bone formation as ALP is secreted as a by-product of osteoblast activity. Significant increase of ALP in all groups (other than THP-1) is an indication of ongoing osteoblast differentiation from hMSCs and increase in their activity over the course of culture. Several work in the literature [27, 37–39] reported that complete hMSC differentiation into osteoblasts takes around four weeks which corroborated with the ALP data. Significantly higher ALP activity in hMSC spheroids (figure 3(A)) demonstrated higher osteoblastic differentiation compared to the heterocellular spheroids and THP-1-only cell clusters. No significant difference was observed between the healthy cohort and GD patient groups at Days 14 and 28—indicating similar differentiation of commercial hMSCs into osteoblasts—corroborating the results from a previous study by Lecourt *et al* [40]. This was conflicting with the data published by Panicker *et al* [23], who demonstrated lower differentiation potential of osteoblasts differentiated from GD patient-derived pluripotent stem cells (iPSCs) with defective bone matrix protein and calcium deposition. This might be the case because in our study, hMSCs were commercially obtained from healthy donors and did not have the genotypic characteristics of a GD patient. This can be considered for our future study, where the bone model will be developed with GD patient-derived iPSCs and PBMCs differentiating into osteoblasts and osteoclasts, respectively—making the model more physiologically-relevant with the cells derived from a single source.

Osteoclastic AP is an enzyme, which is synthesized in abundance by the active osteoclasts during normal and GD-related bone disorders. Previous studies have reported a direct evidence of increased TRAP activity with increased osteoclastic activity and consequently bone resorption [41]. Higher AP activity (figure 3(B)) in the THP-1-only group demonstrated higher densities of macrophages or active osteoclasts as per our hypothesis. Significantly higher AP activity in the GD-severe group compared to the healthy cohort revealed higher osteoclastic activity in GD patients, which was in agreement with previous reports and expression of clinical biochemical markers as well [42]. As expected, negligible ALP and AP activity was detected in THP-1-only and hMSC groups, respectively. It is worthy to note that commercially available THP-1, peripheral blood derived monocytes, were used as a positive control as well. THP-1 cells were observed to form clusters without formation of stand-alone intact spheroids. In such regard, it was not possible to aspirate and bioprint them, so this group was not further considered for the bioprinting related efforts.

**Figure 4.**
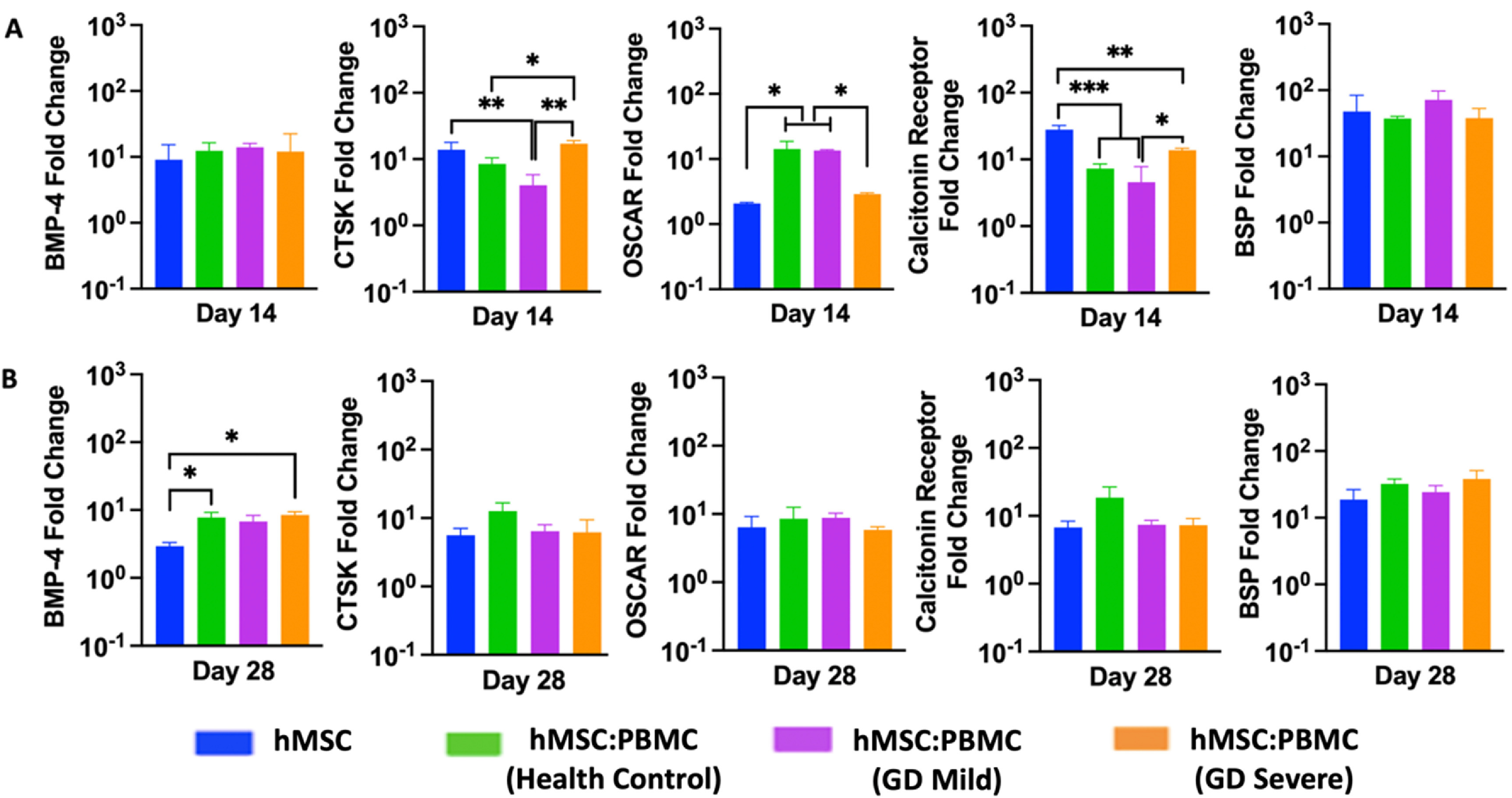
Gene expression data for hMSC:PBMC spheroids at Days 14 and 28. Quantification of BMP-4, BSP, OSCAR, calcitonin receptor, and CTSK gene expressions (*n* = 3, *p < 0.05 **p < 0.01, and ***p < 0.001).

The relative osteogenic gene expression of hMSC-only and hMSC:PBMC spheroids was measured by qRT-PCR on Days 14 and 28. The expression of osteogenic and osteoclastogenic genes including BMP-4, BSP, CTSK, OSCAR and Calcitonin receptor (figure 4) were determined. Although the expression levels of BMP-4 gene were similar for all groups at Day 14, the GD-severe group marker showed a significant increase (∼9-folds) in BMP-4 expression as compared to the hMSCs group (∼3-folds) at Day 28. Interestingly, all markers but BMP-4 showed similar expression with no difference over time at Day 28 for the single spheroids. No significant difference in the expression of both the osteoblastic differentiation markers (early-stage marker BMP-4 at Day 14 and late-stage marker BSP at both Days 14 and 28) in hMSC and all heterocellular spheroids (including the GD patient groups) again provided evidence to no significant alterations of osteoblastic differentiation from hMSCs in GD patients.

### 3.2. Bioprinting of an *in-vitro* GD bone model using hMSC:PBMC heterocellular spheroids

**Figure 5.**
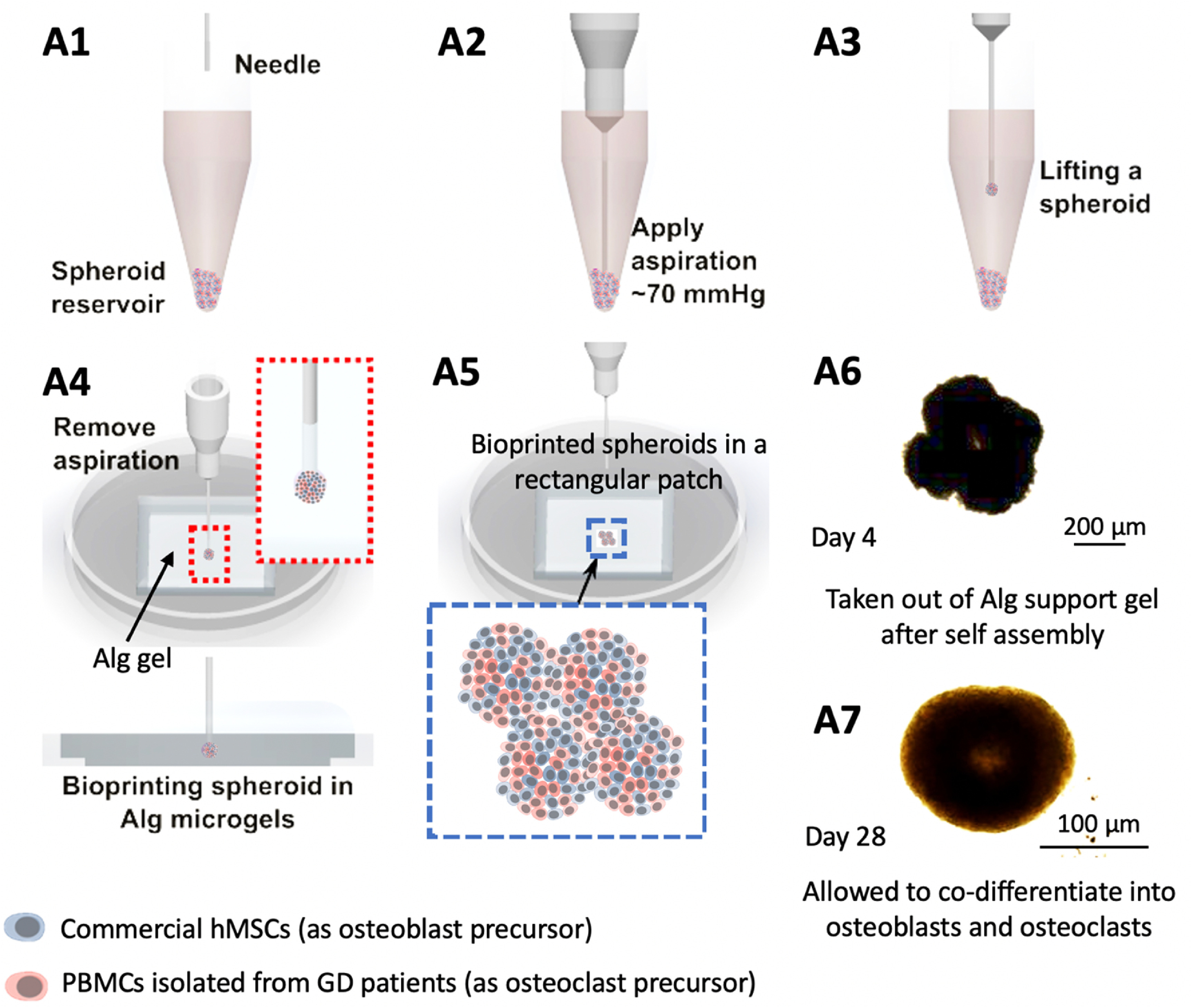
Aspiration-assisted freeform bioprinting process. (A1-A7) A schematic demonstrating the process in a step-by-step manner, where hMSC:PBMC spheroids were lifted and bioprinted into Alg microgels in an iterative manner to form a rectangular patch of tissue.

Bioprinting is an emerging technology, which has the potential to facilitate the development of miniaturized tissue models or even geometrically-complicated volumetric scaffolds with complex spatial heterogeneity, cellular composition, or extra-cellular matrix (like collagen, glycoproteins, or other growth factors or enzymes). Over the past decade, much of the literature has focused on bioprinting of cell-laden hydrogels [43–45]. Even though such attempts have considerably advanced the field, inability to generate physiologically-relevant cell densities and limited intra- or inter-cellular crosstalk generates the need for scaffold-free bioprinting approaches [46–48]. Cellular aggregates, especially spheroids, have been the obvious choice of bioink, because they possess suitable mechanical properties for bioprinting, and enable achievement of physiologically-relevant cell densities. In this regard, we employed aspiration-assisted freeform bioprinting technique as highlighted in figure 5, which enabled aspiration of individual spheroids, and their precise placement one by one in yield-stress gels, like Alg microgels, in close proximity to each other allowing their self-assembly into a tissue patch. Despite single spheroids could also be used as a bone tissue model for GD, we preferred to build an assembled model using spheroids as building blocks, where the assembly was realized after bioprinting through the fusion of spheroids mimicking the bone development process. Even though the bioprinting process was slow, close investigation of fusion of spheroids to form tissues and mimic GD-related bone deformities was valuable for this study. As our goal in this work is to build a GD model, we preferred to construct a structure with four spheroids only. For geometrically more complex architectures, the reader is referred to previous studies [27, 28, 39, 49].

Using Alg microgels, we bioprinted hMSC spheroids and osteogenically inducted them to demonstrate bone tissue formation. To systematically study the influence of the addition of the osteoclastogenically-inducted monocytes from GD patients, we also bioprinted 2:1 hMSC:PBMC (healthy, GD mild and GD severe) spheroids in a rectangular pattern using aspiration-assisted freeform bioprinting (supplementary video 1). As manual placement of spheroids in close proximity to enable fusion and forming larger tissue is not possible inside the yield-stress gel, a non-bioprinted control group was not considered; however, single spheroids were used as a control group. These spheroids were cultured in a cocktail of the individual growth media, mixed in a 2:1 ratio, for 5 d to allow their self-assembly into a bone tissue patch. Thereafter, Alg microgels were decrosslinked and removed using sodium citrate and the bioprinted tissue was cultured for 28 d in a 2:1 cocktail of osteoblast and osteoclast differentiation media.

**Figure 6.**
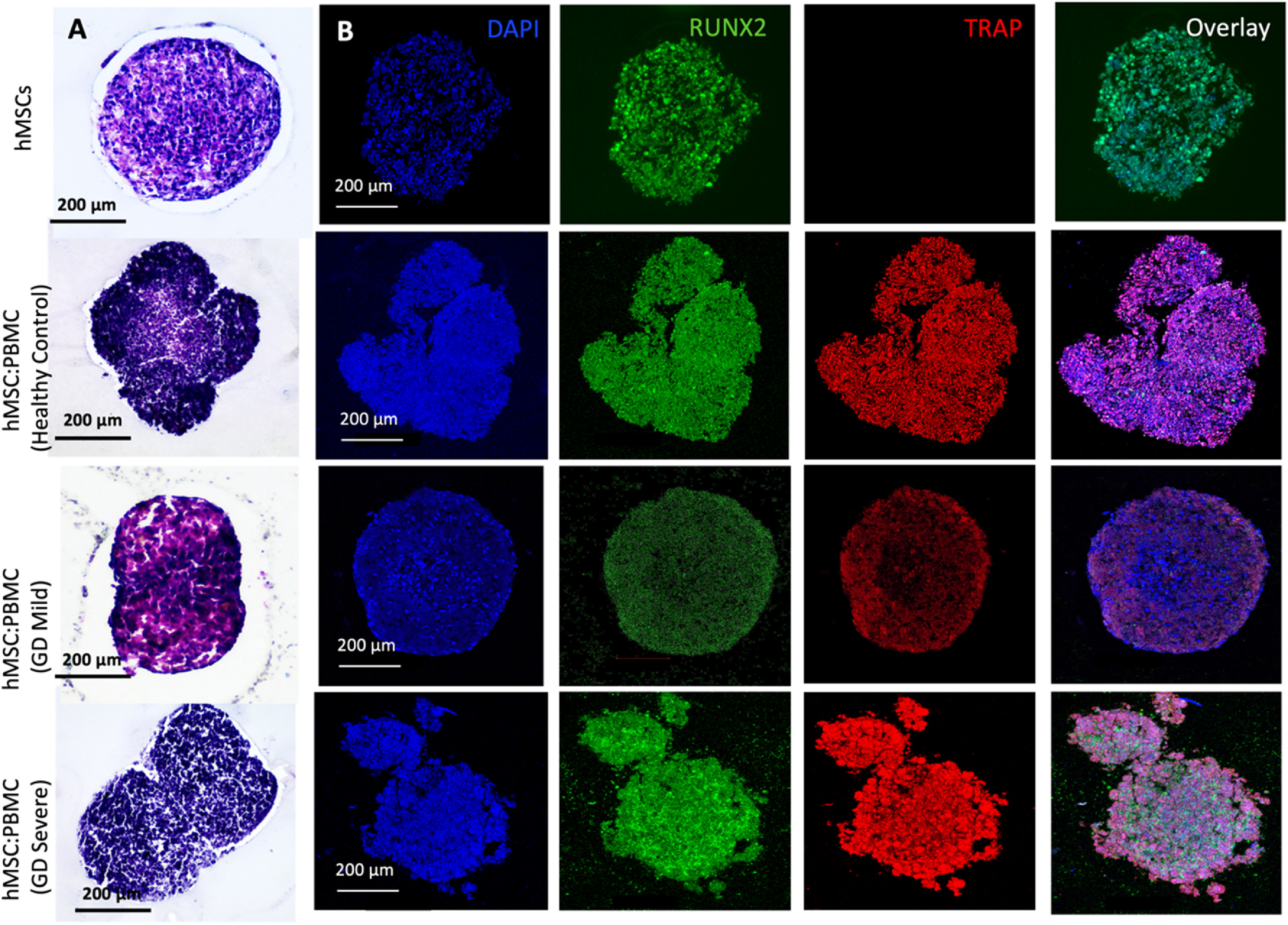
Bioprinting of hMSC:PBMC spheroids to fabricate a bone tissue model for GD at Day 14. (A) H&E and (B) immunostaining for DAPI (blue), RUNX2 (green), TRAP (red) and overlay.

**Figure 7.**
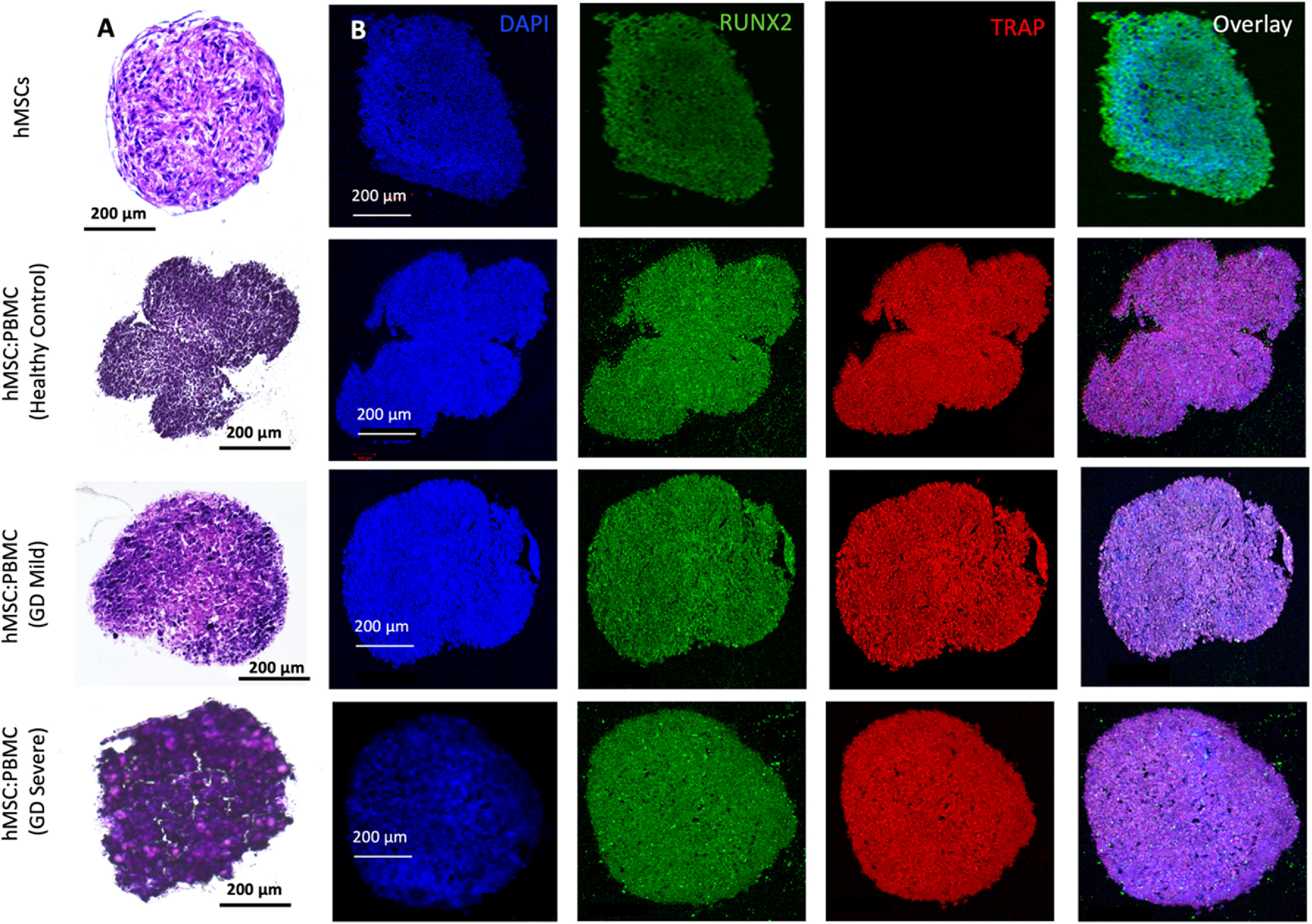
Bioprinting of hMSC:PBMC spheroids to fabricate a bone tissue model for GD at Day 28. (A) H&E and (B) immunostaining for DAPI (blue), RUNX2 (green), TRAP (red) and overlay.

**Figure 8.**
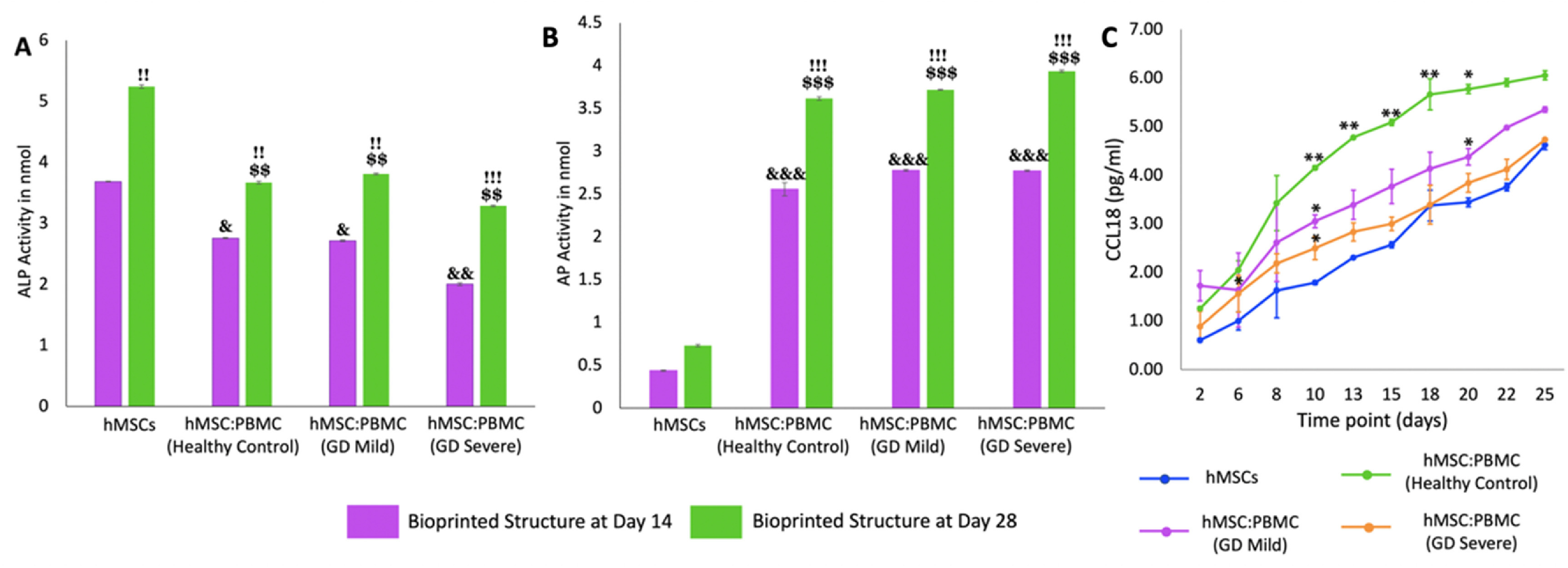
Enzymatic assays for bioprinted hMSC:PBMC spheroids at Days 14 and 28. (A) ALP enzyme activity for hMSCs differentiating into osteoblasts and (B) AP for PBMCs differentiating into osteoclasts. Data were presented as mean ± s.e.m (*n* = 4; &: compared to hMSCs (Day 14) *p* ^&^< 0.05, *p* ^&&^< 0.01, *p* ^&&&^< 0.001; $: compared to hMSCs (Day 28), *p* ^$^< 0.05, *p* ^$$^< 0.01, *p* ^$$$^< 0.001; !: compared to Day 14, *p* ^!^< 0.05, *p* ^!!^< 0.01, *p* ^!!!^< 0.001) (C) CCL18 activity at Day 28. Data were presented as mean ± s.e.m (*n* = 3; *: compared to hMSCs at each time point *p* ^*^< 0.05, *p* ^**^ < 0.01).

H&E and immunostaining (figures 6 and 7) with osteoblastic and osteoclastic specific markers proved that both the hMSC and PBMC-involved constructs showed compact spheroid arrangements. The hMSC group was observed to compact significantly and form a tissue ball (figures 6(A) and 7(A)) over the 28 d culture period. This is in coherence with the previous findings in the literature [49]. The preculture time of spheroids could be modified to optimize the shape retention of the bioprinted structures with a trade-off with the self-assembly potential. Bioprinted hMSC:PBMC spheroids (from the healthy cohort) were observed to maintain the original shape better than the hMSC-only group during the entire course of culture. Thus, it is relevant to conclude that the introduction of PBMCs into hMSC spheroids reduced the tissue compaction, hence contributing to shape retention at Day 28, as shown from the hMSC:PBMC healthy cohort. Similar morphological deformities, as were also observed with single spheroids, were noted in the bioprinted constructs. None of the GD patient groups retained their shape opposed to the healthy cohort and thus turned into a ball shape, which is also often noted with several bioprinting studies, when spheroids are used as building blocks [27, 28, 39, 49]. These results are in cohesion with the skeletal deformities noted in GD patients, depending on the severity of the disease progression. We observed a crumbled paper-like abundant cytoplasm in the GD mild group, whereas the GD severe group demonstrated a more abundance in nuclei with no visible cytoplasm. This indicates that bioprinting did not alter the phenotypes of the cells. Immunostaining images bore evidence to the presence of both osteoblasts and osteoclasts in the heterocellular groups by positive expression of RUNX2 and TRAP, respectively. ALP and AP activity showed higher enzyme activity in all bioprinted constructs compared to the single spheroids (figure 8). We observed a higher expression of both ALP and AP activity at Day 28 compared to Day 14 in the bioprinted constructs, which was also in corroboration to our observation with single spheroids. Compared to the hMSC-only bioprinted group, higher AP activity was observed in all groups with patient-derived PBMCs. No significant difference was observed in the healthy cohort compared to the GD patient groups for ALP and AP activity. Notably, compared to hMSC-only and healthy cohort groups, we observed slight changes in ALP activity in the GD patient groups, still in coherence with our hypothesis of marginal changes in osteoblast differentiation in the GD-patient groups. But when compared to the AP activity, we observed a significant difference in the GD groups compared to the healthy cohort.

Next, we analyzed the functional behavior of GD versus healthy control macrophages/osteoclasts in the bone constructs. In GD, the elevation of inflammatory molecule CCL18 in blood indicates activation of macrophages and/or Gaucher cells. Overall, CCL18 is a macrophage inflammatory marker with expression and secretion primarily by cells of myeloid origin. Expression of CCL18 increased during monocyte/macrophage/osteoclast differentiation [50]. The measurement of CCL18 in cell culture media revealed increased CCL18 secretion over time (figure 8(C)). The increase in CCL18 levels over the culture period was because of the increased cell proliferation as in corroboration from the literature [51]. Mono-cultured hMSCs differentiated into osteoblasts showed similar but much lower production of CCL18 than co-cultured hMSCs and PBMCs. The healthy control group showed the most abundant CCL18 secretion. These data indicated that cocultured hMSC:PBMC spheroids to form 3D bone tissue could differentiate PBMCs into functionally normal macrophages-osteoclasts and induced the secretion of inflammatory molecules.

**Figure 9.**
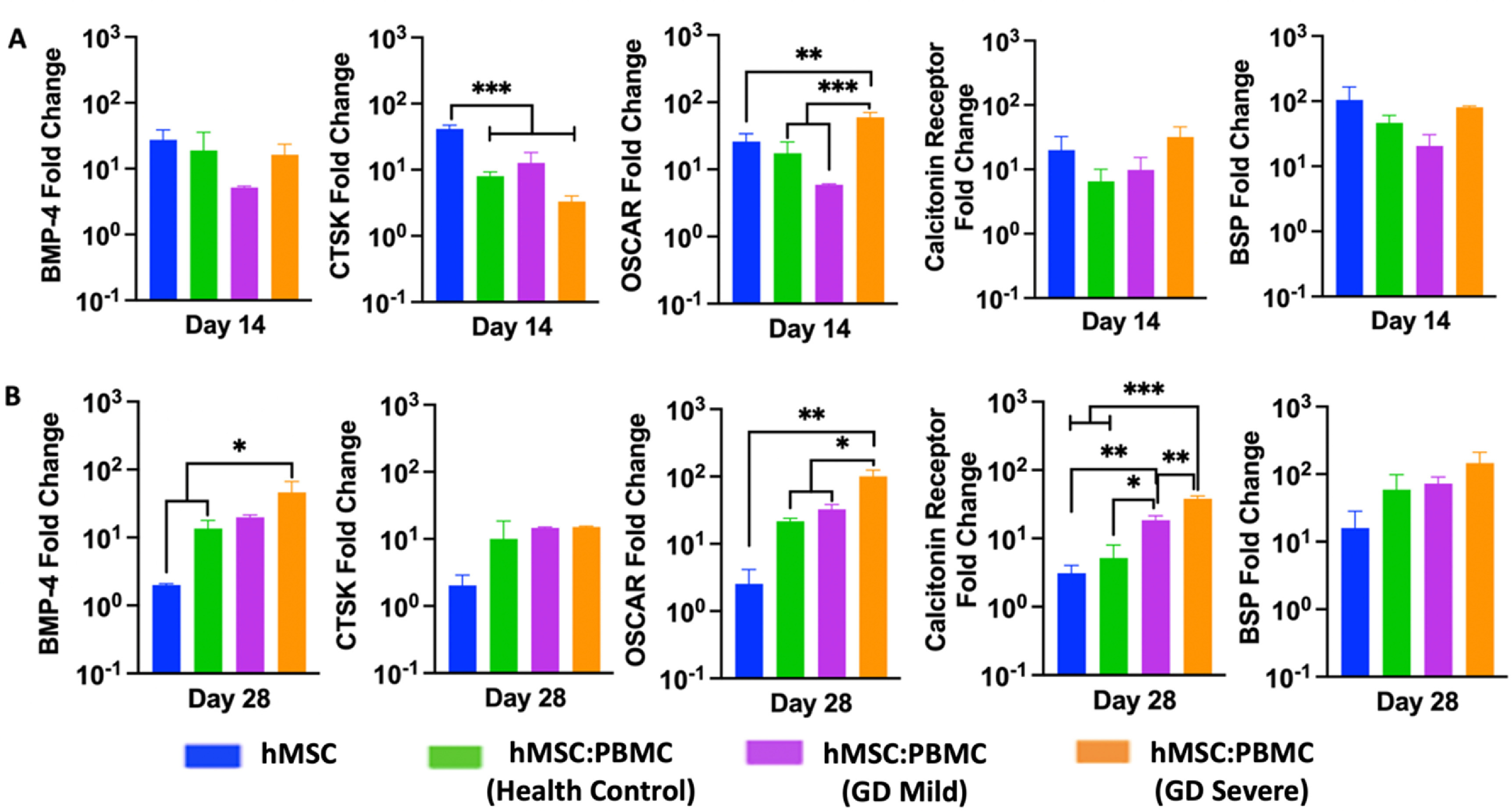
Gene expression data for the bone tissue model bioprinted using spheroids. Quantification of BMP-4, BSP, OSCAR, calcitonin receptor, and CTSK gene expressions at Days 14 and 28 (*n* = 3, *p < 0.05 **p < 0.01, and ***p < 0.001).

The relative osteogenic gene expression (BMP-4, BSP, Calcitonin receptor, OSCAR, and CTSK) of bioprinted constructs composed of hMSC-only or hMSC:PBMC heterocellular spheroids was measured on Days 14 and 28 (figure 9). Although the bioprinted groups showed no significant difference in BMP-4, Calcitonin receptor and BSP at Day 14, the GD-severe group exhibited significantly increased expression levels for those markers (BMP-4: ∼46-folds, Calcitonin Receptor: ∼38-folds, and BSP: ∼147-folds) at Day 28. At Day 28, bioprinted constructs of the GD groups (mild and severe) showed higher level expressions of osteogenic markers as compared to hMSCs-only and healthy cohort groups. While the hMSCs-only group exhibited significant difference for CTSK gene as compared to GD healthy, GD mild, and GD severe groups at Day 14, no significant difference was observed among those groups for the expression of CTSK gene at Day 28. There was a significant decrease in the expression levels for BMP-4, CTSK, OSCAR, Calcitonin Receptor, and BSP in hMSCs group at Day 28 as compared to those at Day 14 with a fold-decrease of ∼13.8, ∼20.3, ∼10.2, ∼6.4, and ∼6.5, respectively. Compared to other groups, significantly higher OSCAR expression was observed in the GD severe group, as was reported before [52]. The GD patient groups demonstrated an increasing trend over time in osteogenic and osteoclastic gene expression over time compared to the control groups. This indicates a delay in both osteoblast and osteoclast differentiation with the incorporation of GD patient-derived PBMCs in the bioprinted groups. qPCR data might not be directly correlated to the enzymatic assay activity. The differences between qPCR and enzymatic activity assays could be attributed to a different cellular mechanism involved—causing changes in protein degradation, influencing cellular enhancers or promoters upstream of the regulating genes without changes in transcriptional processes. Alongside, determination of different cell types (other than osteoblasts and osteoclasts, such as undifferentiated hMSCs and monocytes) present in the fused tissue possibly by RNA sequencing at different time points will be required to corroborate qPCR data with the enzymatic activity data, which is out of the scope of the current study. On comparing the bioprinted groups with the single spheroid groups, we showed that bioprinting of spheroids with GD supported higher osteogenic marker expressions over time compared to single spheroids, also observed in enzymatic activity. Expression of the osteoclastogenesis markers OSCAR and CTSK indicated enhanced osteoclast activation in bioprinted groups with GD as compared to hMSCs-only group at Day 28, as would be expected with increased osteoclast activity in GD severe groups [53].

Overall, we aimed to fabricate an *in-vitro* bone model to understand GD. Several studies investigating GD has focused on osteoclast- or osteoblast-only models and induction of GD by chemical agents, mostly with the use conduritol-B-epoxide (CBE) [54]. Although the individual osteoblast or osteoclast cellular models are far from the recapitulation of GD in bone abnormalities, the use of CBE works by inhibition of the glucocerebrosidase enzyme activity, and has considerable phenotypic similarity to GD by enabling lipid accumulation Even though the use of CBE provides an inexpensive and quick way to understand the molecular mechanism of GD pathology, limited studies have been performed on the demonstration of phenotypic relevance and importance of dosage of CBE on bone abnormalities [40]. On the other hand, generation of animal models that faithfully recapitulate the genotypes and phenotypes of GD has been more challenging than anticipated with several studies reporting death or phenotypic symptoms mismatch with point mutation in the glucocerebrosidase enzyme in mice models [55]. In such regard, this study becomes relevant because it introduces a human bone tissue model using high cell density spheroids developed from co-culture of hMSCs-derived osteoblasts and PBMCs-derived osteoclasts (isolated from GD patients) for testing of novel therapeutics for GD.

## 4. Conclusion

In this study, we demonstrated the first bioprinted *in-vitro* GD model, which composed of heterocellular hMSC:PBMC spheroids co-differentiated to form osteoblasts and osteoclasts, respectively. PBMCs were isolated from three different categories of clinical samples—healthy cohort and GD mild and GD severe patients. Bioprinting of spheroids enabled their fusion to form a rectangular construct with native-like cell density, phenotypic relevance to GD skeletal abnormalities and expression of both osteoblast- and osteoclast-related specific markers. Our results indicate that the proposed *in-vitro* bone model for GD has the potential to become a prospective disease platform demonstrating physiologically- and phenotypically-relevant symptoms for GD for enabling novel therapeutics. In the future, we plan to utilize the 3D bioprinted GD model for screening of some of these novel therapeutics.

## Data Availability

All data that support the findings of this study are included within the article (and any supplementary files).
